# Sewage Sludge ZnCl_2_-Activated Carbon Intercalated MgFe–LDH Nanocomposites: Insight of the Sorption Mechanism of Improved Removal of Phenol from Water

**DOI:** 10.3390/ijms21051563

**Published:** 2020-02-25

**Authors:** Nuhu Dalhat Mu’azu, Mukarram Zubair, Nabeel Jarrah, Omar Alagha, Mamdouh A. Al-Harthi, Mohammed H. Essa

**Affiliations:** 1 Department of Environmental Engineering, College of Engineering, Imam Abdulrahman Bin Faisal University, P.O. Box 1982, Dammam 31451, Saudi Arabia; mzzubair@iau.edu.sa (M.Z.);; 2 Department of Chemical Engineering, Mutah University, Karak 61710, Jordan; 3 Department of Chemical Engineering, King Fahd University of Petroleum & Minerals, Dhahran 31261, Saudi Arabia; 4 Center of Research Excellences in Nanotechnology, King Fahd University of Petroleum & Minerals, Dhahran 31261, Saudi Arabia; 5 Department of Civil Environmental Engineering, King Fahd University of Petroleum & Minerals, Dhahran 31261, Saudi Arabia

**Keywords:** sludge-activated carbon, MgFe layered double hydroxide, nanocomposite materials, phenol aqueous uptake, mechanistic studies, reusability performance

## Abstract

This work reports the synthesis of new layered double hydroxide (LDH) composites using sewage-based ZnCl_2_-activated carbon (AC) intercalated with MgFe (AC-MgFe-LDH) and an evaluation of their adsorptive performance for phenol removal from water. The effect of the AC loading on the final properties of synthesized composites was investigated via various characterization techniques. The results showed efficient decoration at 0.1–0.25 g AC loading within the layers of AC–MgFe composites LDH, which was reflected in the higher surface area (233.75 m^2^/g) and surface functionalities (OH, NO_3_, C-O-C, and MMO) yielding a significant improvement of the phenol removal efficiency. However, at higher contents, AC loading led to the breakage of the LDH structure and agglomeration, as indicated by the deterioration in the textural and structural properties. The isotherm and kinetic data were well fitted by the Langmuir and pseudo-second-order model, respectively, with a maximum obtained monolayer adsorption capacity of 138.69 mg/g. The thermodynamics results demonstrated that phenol adsorption is an endothermic process. The sorption mechanism of phenol molecules on the AC–MgFe composite was governed by chemical bonding with OH, C=O, and MMO groups and pore diffusion via π–π interactions. Superior phenol removal with excellent recyclability up to five cycles of the new AC–MgFe composite suggested its use as a potential adsorbent for effective phenol removal from water and wastewater streams.

## 1. Introduction

During the last two decades, rapid industrial development has induced significant production of organic waste, causing severe water pollution and a negative impact on the environment [1]. Among organic contaminants, phenol is considered a highly hazardous pollutant due to its serious harm on living organisms even at moderate exposure (low concentration) [2]. According to the US environmental protection agency, the maximum permissible limit of phenol in wastewater and water supplies is 0.1 mg/L and 1 µg/L. Phenol-contaminated wastewater is a product of various industry processes, including polymer, drugs, dyes, petrochemical, and pesticide industries [3]. Phenol is a potential carcinogen, with its degradation resulting in carcinogenic and toxic byproducts [4]. Phenol causes damage to the kidney and liver, tissue erosion, and disorder of the nervous system [5]. Therefore, the direct release of phenol-contaminated industrial effluent has been strictly prohibited due to its serious and long-term consequences on human health and the environment. As such, it is essential, to develop effective and sustainable remediation and discharge of organic wastewater systems for a safe and sustainable environment.

Various physiochemical techniques have been utilized for the treatment of phenol-containing wastewater, including precipitation, oxidation, membrane filtration, and adsorption. Among all of them, adsorption is considered a highly effective and suitable process for the removal of phenols from industrial effluents [6] due to its simple design, user-friendliness, low operation and maintenance cost, and high remediation performance [7]. In the last decades, various adsorbents, such as carbon materials [8,9], clays [10], polymers [11], and silica [12], have been investigated for their adsorption of phenolic contaminants. However, due to its high production cost, low adsorption capability for different pollutants, limitation in industrial effluents’ pH range, and low reusability, increasing interest has focused on developing new sustainable and eco-economical hybrid adsorbents that exhib a high removal and excellent reusability performance for various pollutants.

Activated carbon (AC) is a widely used adsorbent for the remediation of contaminated organic wastewater due to its excellent surface characteristics (surface area and porous structure) and abundant oxygen functionalities [6,13]. For example, AC derived from rattan sawdust exhibited high phenol removal, with a maximum adsorption capacity of 149.25 mg/g [14]. Low-cost ZnCl_2_–AC produced from tea waste revealed an outstanding adsorption of phenol (142.9 mg/g) [15]. Recently, Ju Sun et al. [16] reported the increasing oxidation degree of AC resulted in a decrease in sorption of phenol and the mechanism was dominated by π–π interactions. In our previous work [17], we produced ZnCl_2_-activated carbon from sewage sludge with different chemical activation conditions and obtained adsorption of phenol of 9.91 mg/g (under a competitive environment with catechol and resorcinol) at a ZnCl_2_ -sludge ratio between 1–1.5, temperature of 400 to 700 °C, and activation time of 30–36.74 min, with a percent yield of 81.5%. However, the adsorption capacity increased up to 20.4 mg/g for single phenol adsorption.

Layered double hydroxides (LDHs), and its composites, are a promising adsorbent for various organic and inorganic pollutants [18]. Their excellent ion exchangeability, high surface area, and lower toxicity with versatile composition are potentially attractive for effective water and wastewater treatment. LDH hybridization, i.e., the coupling or decoration of LDH layers into various materials, including carbon materials (graphene, carbon nano tube (CNT), biochar), polymers (starch and chitosan, etc.), and others, resulted in extremely promising materials, exhibiting enhanced surface and structure characteristics [18,19]. Recently, several LDHs and its hybrids have been explored for the removal of phenols from water bodies. For instance, porous NiAl–LDH nanoparticle-modified sodium citrate showed adsorption of p-nitrophenol, with an adsorption capacity 77.7 mg/g [20]. F. T Zompantzi et al. [21] reported 95% phenol degradation using ZnAl metal oxides. Highly stable Al/Fe pillared clay catalyst was synthesized and showed efficient remediation of phenol and total organic carbon (TOC ) from aqueous solutions [22]. MgAl precursor prepared by simple dry milling of Mg and Al salts exhibited a high sorption capacity of 82.5 and 356.4 mg/g for phenol and p-nitrophenol, indicating excellent adsorbent for the treatment of phenolic compounds from the aqueous phase. Calcined MgAl-decorated single-walled carbon nanotubes (SWNTs) revealed an extremely outstanding affinity for phenol and 4-chloro phenol, with remarkable reusability performance for up to 10 cycles. The author reported the improvement in the adsorption capacity of LDH is due to the coupling of SWNT and calcination [23]. Previous studies demonstrated that the modification of LDH could lead to enhancement of active binding sites, such as surface oxygen functional groups, and resulted in a substantial increase in the uptake of phenolic compounds. The most recent detailed literature review undertaken revealed that the synthesis of high-performing adsorbents via intercalation of sewage-based AC into the layers of LDH has not yet been reported. Since both sewage-based AC and LDH are good adsorbents with a high affinity for phenols, their hybridization at appropriate ratios was speculated to result in new composite materials possessing improved adsorptive performances compared to the parent materials as well as many other adsorbents.

Accordingly, the objective of this study was to fabricate ZnCl_2_-activated carbon (AC) decorated MgFe–LDH composites at different compositions of AC (0.1–0.5 g) via the co-precipitation technique. The synthesized composite materials were utilized as adsorbents for the removal of phenol from the water phase. The composites were fully characterized by FTIR, XRD, SEM, and N_2_ adsorption-desorption measurement before and after phenol adsorption to deeply assess the changes in characteristics after the addition of AC and phenol sorption mechanisms. Additionally, the influence of pH, contact time, initial phenol concentration, temperature, adsorption equilibrium, and kinetics were studied using batch mode experiments.

## 2. Results

### 2.1. Characterization of AC–MgFe Composites

**Figure 1:**
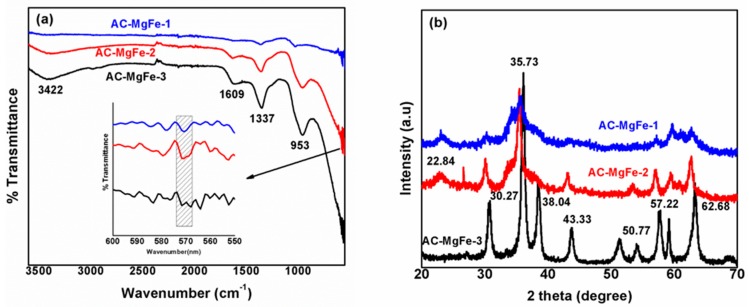
FTIR spectra of AC–MgFe composites (**a**), XRD pattern of AC–MgFe composites (**b**).

The FTIR spectra of AC–MgFe composites are displayed in Figure 1a. Noticeably, the spectra of all three composites exhibited characteristic bands of both AC and MgFe, as indicated in Figure S1a,b, respectively, [18,24] with observable variations in band intensities. The broad and strong peak at 3422 cm^−1^ corresponds to the stretching vibration of hydroxyl groups (O-H), associated with the water molecules and hydrogen bonding within the interlayers of the AC–MgFe composites. Similarly, the sharp peak at 13,857 cm^−1^ was attributed to the NO_3_^2−^ ions’ vibration in the layers of MgFe LDH (Figure S1b), which was shifted to a lower wavenumber of 1337 cm^−1^ in the AC–MgFe composites (Figure 1a) [25]. Two strong and sharp peaks at 1609 and 953 cm^−1^ were also observed, which are attributed to the stretching vibrations of (C=N or C=O) and (C-O or C-C) groups, respectively, which correspond to surface functionalities of AC (Figure S1a). Besides, the presence of a peak at around 571 cm^−1^ might be attributed to the formation of mixed metal oxides (MMOs) (M= Mg, Fe). The AC–MgFe-2 composite showed a strong and broad peak of MMO compared to other AC–MgFe composites. Markedly, the spectra of composites (AC–MgFe-2 and AC–MgFe-1) consisted of a lower content of AC (0.25 and 0.1 g), respectively, which indicated stronger characteristic band intensities compared to the higher AC content composite (AC–MgFe-3). The FTIR results demonstrated the effective formation of AC–MgFe composite, exhibiting abundant oxygen-containing functionalities (OH, NO_3_, C=N, C=O, C-O, and MMO), and thereby exhibiting good adsorbent characteristics for the uptake of pollutants from the aqueous phase [6].

The XRD patterns of AC–MgFe composites are shown in Figure 1b. The sharp diffraction peaks at 2° = 18.54° 30.27°, 35.73°, 38.04°, 43.33°, 57.22°, and 62.68 ° in the XRD patterns of the AC–MgFe-3 composite are characteristics of MgFe LDH nanoparticles [26]. Similarly, the broad diffraction peak at 22.84° in the XRD patterns of AC–MgFe-2 and AC–MgFe-3 was attributed to the (002) plane of graphitic carbon and corresponds to the diffraction peak of AC. Noteworthy, both AC–MgFe-3 and AC–MgFe-2, which are associated with a lower content of AC (0.1–0.25 g), revealed characteristic diffraction peaks of MgFe, indicating excellent crystallographic-structured MgFe layers, and facilitating better intercalation without damaging them. Whereas in AC–MgFe-1 and AC–MgFe-2, the lower peaks’ symmetries and absence of some of the peaks suggest a poor crystalline structure attributed to higher loading of AC compared to AC–MgFe-3, which led to agglomeration or stacking onto the layers of LDH [23,27].

**Figure 2:**
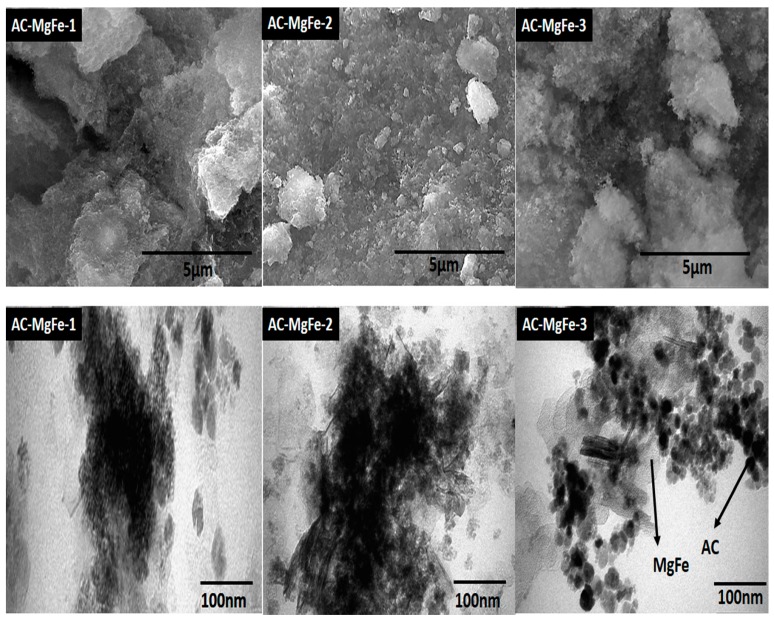
SEM and TEM images of AC–MgFe composites.

SEM and TEM analyses were used to evaluate the surface morphology and dispersion of AC into the LDHs structure. Figure S2 shows an SEM image of the AC, showing fluffy, amorphous, and cracking surface morphology exhibiting smooth granular particles with a 500–1000 nm size. In Figure 2, the morphology of all AC–MgFe composites shows a rough and porous surface. For AC–MgFe-1, the surface is comprised of discrete patterns with a highly porous and amorphous structure associated with the presence of high loading (0.5 g) of amorphous AC. However, the SEM image of AC–MgFe-2 indicates a coarse surface, exhibiting particles of varied sizes. This may be associated with better dispersion of AC nanoparticles within the layers of the MgFe LDH. The dispersion of AC onto the AC–MgFe composites was further supported by TEM analysis (Figure 2). The TEM of AC–MgFe-3 showed partial dispersion of AC nanoparticles associated with the lower content of AC (0.1 g) into the composite. For AC–MgFe-2, higher decoration of AC into the layers of the MgFe led to an improvement in the surface, textural, and crystalline characteristics of the composite and promoted enhanced uptake of phenol molecules from the aqueous phase. However, the higher agglomeration and stacking into the LDH layers, leading to poor crystallinity and oxygen functionalities, was attributed to the presence of high contents of AC, especially in the AC–MgFe-1 (0.5 g), as confirmed by XRD and FTIR analysis, respectively. As the XRD crystallization for AC–MgFe-1 and AC–MgFe-2 was not very good (compared with that of AC–MgFe-3) as highlighted earlier, reliable crystallite calculation was not possible for these two adsorbents. As such, the Debby Scherrer formula [28] was used to calculate the DXRD only for the AC–MgFe-3, which was found to be around 30 nm (33 ± 3 nm).

**Figure 3:**
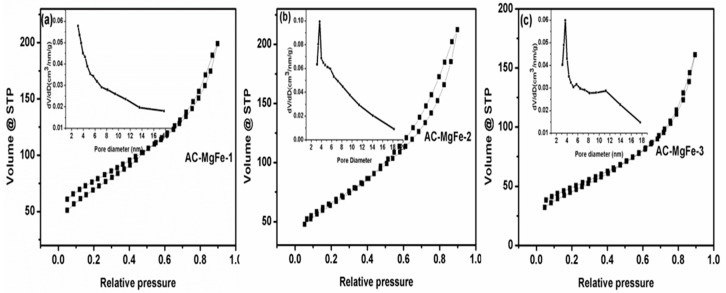
N_2_ adsorption/desorption for (**a**) AC-MgFe-1 (**b**) AC-MgFe-2 and (**c**) AC-MgFe-3 AC–MgFe composites.

The textural characteristics (specific surface area, pore volume, and pore width) of the AC–MgFe composites were obtained by N_2_ adsorption-desorption isotherms (Table 1), and their corresponding adsorption-desorption plots are displayed in Figure 3a–c. The shape of adsorption-desorption isotherm of all three AC–MgFe composites are hysteresis loops of Type IV, confirming predominantly mesoporous solids. As seen in Table 1, a significant increase in the surface area was observed from 168.92 to 233.76 mg/g as the AC content in the layers of MgFe was raised from 0.1 to 0.25 g, respectively, a significant improvement compared to virgin MgFe as reported previously [29]. However, further loading of AC to 0.5 g (AC–MgFe-1) yielded an insignificant increase in the surface area (Table 1), which was ascribed to the agglomeration of AC nanoparticles in the AC–MgFe-1 composite as confirmed by the TEM analysis. The as-produced AC–MgFe composites exhibited a higher surface area compared to pristine MgFeLDH [30] and other previously studied magnetic adsorbents [31]. This indicated that coupling of the sewage sludge AC with MgFe is a highly promising and sustainable approach to significantly improve the surface area, which may result in enhanced removal of pollutants from wastewater. Likewise, the pore volume increased from 0.21 to 0.27 cm^3^/g when the AC content in the layers of MgFe–LDH increased from 0.1 to 0.5 g, which was attributed to the diffusion of AC nanoparticles within the pores of MgFe–LDH. Moreover, the pore size distribution of AC–MgFe composites showed a maximum volume at a pore diameter of 3.9 to 5.4 nm, indicating mesoporous characteristics, and is highly favorable for the penetration of phenol molecules in pores greater than 1 nm [32].

### 2.2. Phenol adsorption Performance (q_e_) of AC–MgFe Composites at Varied Initial pH

**Figure 4:**
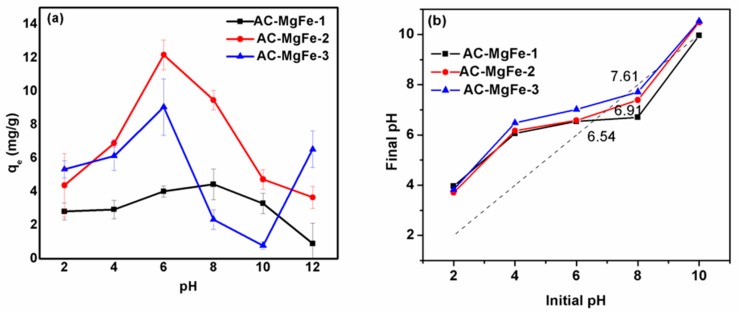
(**a**) Adsorption performance of phenol onto AC–MgFe composites at varied initial pH (**b**) point of zero charge of AC–MgFe-1, AC–MgFe-2 and AC–MgFe-3 composites.

Solution pH is one of the key influential adsorption parameters that has a direct impact on the surface chemistry of both adsorbent and adsorbate and thereby may cause a drastic change of the adsorbent adsorption efficiency. The influence of the initial phenol solution pH onto the sorption capacity of AC–MgFe composites was investigated at a varied pH range 2–12, and other parameters were kept constant at an initial concentration of 20 mg/L, dosage of 5 mg, contact time of 24 h, rpm of 275, and temperature of 25 °C. The results are depicted in Figure 4a. It can be seen that the phenol solution pH significantly alters the sorption performance of all the three synthesized AC–MgFe composites. For instance, at the acidic pH range (2–4), the composites showed a very low sorption capacity of phenol. With an increase in the pH value from 2 to 6, there was a substantial improvement (almost double) in the sorption capacity of phenol from 4.12 and 5.23 mg/g to 12.48 and 9.16 mg/g for AC–MgFe-2 and AC–MgFe-3, respectively. A further increase in the solution pH to 12 led to a reduction in the nanocomposites’ capacities for phenol uptake. However, in the case of the AC–MgFe-1 composite, an increase of the solution pH from 2 to 8 showed a gradual rise of the sorption capacity from 3.13 to 4.08 mg/g and decreased with a further increase of the pH to 12. The change in the sorption capacity of the composites with increasing pH values (2–6) can be explained by considering the surface chemistry of the AC–MgFe composites as illustrated by the estimated point of the zero charge (pH_PZC_) of each composite using the pH drift method [33]. The pH_PZC_ of AC–MgFe-1, AC–MgFe-2, and AC–MgFe-3 was found to be 6.54, 6.91, and 7.61, respectively (Figure 4b). Therefore, for all the studied composites at pH range 2–4 < pH_PZC_, the surfaces of the composites were entirely in the protonation state. This indicates that there may be strong electrostatic repulsion between the composite surfaces and phenol molecules, resulting in lower uptake of phenol onto the composite surface. As the pH was increased to 6 (Figure 4a), there was a significant reduction of positively charged adsorption sites, which was demonstrated in the linear increase in the composites’ adsorption capacity for AC–MgFe-2 and AC–MgFe-3. A similar behavior was reported by Zhang et al. [23] for the removal of phenols by calcined MgAl/SWCNT composites. At pH > 6, there was a gradual transformation of the composites’ active sites to negative, which may induce electrostatic repulsion between the AC–MgFe composites and phenol molecules, thereby resulting in a reduction in phenol removal as the pH increases above 6. The maximum sorption capacity of phenol onto the AC–MgFe composites was obtained at pH 6, which was selected for later adsorption experiments. In addition, AC–MgFe-2 showed a higher removal performance of phenol compared to AC–MgFe-1 and AC–MgFe-3. The higher uptake capacity of AC–MgFe-2 is associated with the effective intercalation of AC onto interlayers of MgFe LDH, leading to enhanced surface, textural, and structural characteristics as confirmed by FTIR, XRD, BET, SEM, and TEM analysis, respectively. Therefore, AC–MgFe-2 was chosen for further phenol adsorption investigations.

## 3. Discussion

### 3.1. Influence of AC–MgFe-2 Dosage and Adsorption Contact Time on Phenol Uptake, q_e_

**Figure 5:**
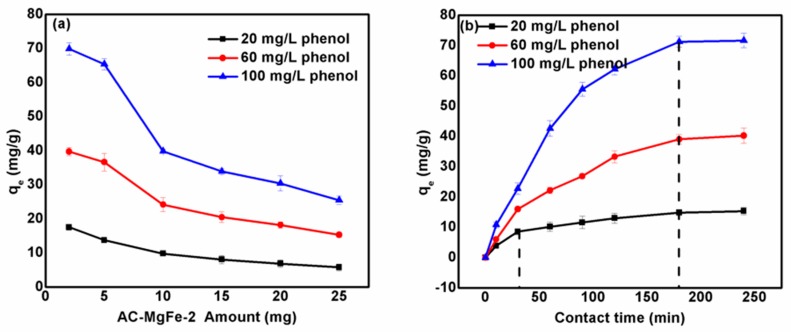
(**a**) Effect of dosage and (**b**) adsorption contact time on the sorption uptake of phenol by AC–MgFe-2 composite.

The influence of the AC–MgFe-2 dosage at different phenol concentrations (20, 60, and 100 mg/L) on the adsorption efficiency (q_e_) of phenol was investigated by varying the dosage from 2–25 mg at pH 6, contact time of 24 h, and temperature of 25 °C. The results are depicted in Figure 5a. Overall, the sorption capacity of phenol increased with an increasing initial concentration at a fixed amount of AC–MgFe-2. In detail (Figure 5a), as the initial concentration was increased from 20–100 mg/L, the q_e_ substantially increased, which was more pronounced at a lower adsorbent dosage (2–5) mg. At a low phenol concentration (20 mg/L), the q_e_ (mg/g) of AC–MgFe showed a gradual decline as the amount increased from 2–15 mg. However, at higher phenol levels (60 and 100 mg/L), a measurable reduction of about 32.22% and 39.42% of q_e_ from 36.66 mg and 65.42 mg/g to 24.21 and 39.87 mg/g was observed, when the AC–MgFe dosage was raised from 5 mg to 10 mg, respectively. At a fixed AC–MgFe dosage, the availability of active binding sites and contributed surface functionalities were also fixed. Therefore, increasing the phenol concentration involves the addition of more phenol molecules that constantly occupied by the free active sites and facilitates competitive adsorption. However, a reduction in q_e_ with an increase in the AC–MgFe-2 dose involves the provision of more active binding sites, which were not utilized due to limited phenol molecules, thus resulting in a decline in the uptake capacity.

Figure 5b displays the change in the qe (mg/g) of phenol at varying contact times (10–240 min), pH 6, concentration of 20–100 mg/L, and temperature of 25 °C. It was found that the adsorption efficiency of phenol onto AC–MgFe increased with increasing contact time. In detail, at all phenol concentrations (20–100 mg/L) nearly 70–80% phenol removal was achieved at 60–120 min, respectively. After that, the removal rate decreased and eventually reached equilibrium at nearly 180 min. The fast adsorption within the first 60–90 min is attributed to the availability of active binding sites (surface functionalities), which were then progressively saturated, resulting in a reduction in the adsorption rate. Moreover, at any fixed contact time, compared to the low phenol concentration (20 mg/L), the q_e_ value of phenol onto AC–MgFe-2 was not only found to be greater in higher phenol (100 mg/L) solution but a significant increase was also observed from 10.88 to 71.24 mg/g with an increasing contact time from 10 to 180 min. The higher concentration implies more phenol molecules in the solution, which requires additional time to completely adsorb in the adsorbent surface and thereby induce better interactions (mass transfer) compared to low phenol levels.

### 3.2. Phenol Adsorption Kinetics

**Figure 6:**
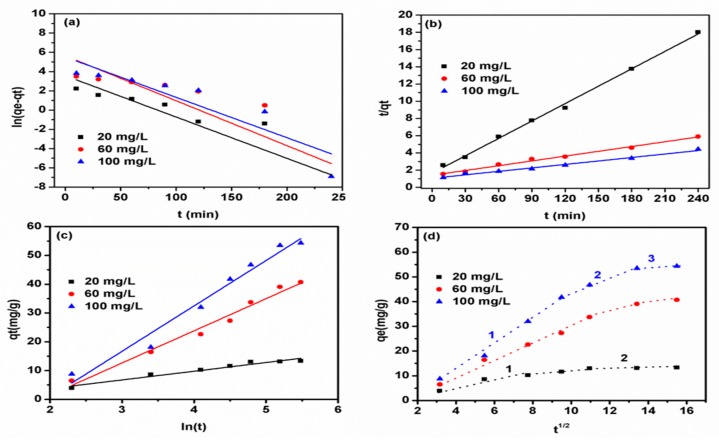
(**a**,**b**) Linear plots of the pseudo-first and second-order model and (**c**,**d**) the elovich and intraparticle diffusion model for the adsorption of phenol on AC–MgFe-2 composites at the 20, 60, and 100 mg/L phenol concentration, respectively.

To further classify the interactions involved in the phenol–AC-MgFe adsorption system (physical or chemical) and the rate-limiting step, the experimental kinetic data were fitted to four kinetic models: The pseudo-first-order, pseudo-second-order, Elovich, and intraparticle diffusion models. The obtained plots using linear kinetic model equations are displayed in Figure 6a–d and their estimated parameters with corresponding R^2^ are summarized in Table 2. Under all tested concentrations, the pseudo-second-order model showed higher R^2^ values (0.987–0.997) compared to pseudo-first-order (0.731–0.836), elovich (0.95–0.97), and intraparticle diffusion (0.79–0.96) models. This indicates that sorption of phenol onto AC–MgFe-2 was more appropriately represented by the pseudo-second-order kinetic model.

Moreover, the increase in the initial concentration led to an enhancement in the adsorption rate (h) from 0.60 to 0.94 mg/g-min, improvement in the gradient pressure between phenol solution and AC–MgFe^-2^, facilitating an increased mass transfer rate at the solid–liquid interface [34]. To further evaluate the actual rate-limiting step, the kinetic data was fitted onto an intraparticle diffusion model (Figure 6d). As shown in Figure 6d, all the plots at the different studied phenol concentrations do not intercept through the origin and involve three distinct adsorption stages. The first stage (fast adsorption rate) is associated with the external surface adsorption involving an interaction of phenol molecules with surface functionalities (OH, C=O, C-O and MMO) via chemical, ion exchange, and π–π interactions. The second stage indicates the pore diffusion of phenols within the interior surface of AC–MgFe-2. The third region describes the equilibrium stage reached due to the saturation of active sites in the AC–MgFe-2. This further demonstrates that the adsorption of phenol molecules on AC–MgFe mainly controlled by multiple mechanisms (physiochemical interactions) and not solely controlled by intraparticle diffusion.

### 3.3. Phenol Adsorption Isotherms

**Figure 7:**
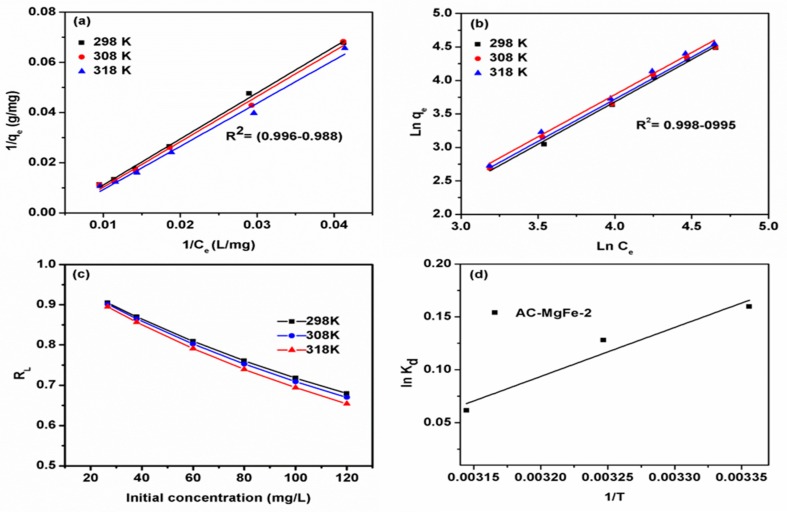
Linear isotherm models (**a**) Langmuir and (**b**) Freundlich and (**c**) R_L_ phenol removal onto AC–MgFe-2 composite and (**d**) plot of Ln Kd vs. 1/T for estimation of the thermodynamic parameters.

Figure 7a,b displays the linear plots of two isotherm models (Langmuir and Freundlich), respectively, applied to the experimental data of phenol adsorption by AC–MgFe-2 at pH 6, contact time of 180 min, and temperatures of 25–45 °C. The calculated parameters, along with the linear regression coefficient (R^2^) and root mean square error (RMSE), are listed in Table 3. As indicated in Figure 7 and Table 3, the equilibrium data for all the studied temperatures fitted both the employed isotherm models well based on the values of R^2^ near to unity (0.992–0.996). However, the error function (RMSE) was found to be smaller for the Langmuir model (0.001–0.002) than that of the Freundlich model (0.046–0.047). Thus, the Langmuir model described the phenol–AC–MgFe adsorption system better, suggesting a monolayer coverage of phenol molecules onto the homogenous AC–MgFe-2 composite surface. Besides, the maximum theoretical adsorption capacity of 138.69 mg/g of the AC–MgFe-2 composite reveals a relatively higher phenol uptake capacity than previously reported biochar composites (Table 4). Additionally, the values of R_L_ are <1 at all studied concentrations (Figure 7c), demonstrating favorable adsorption of phenol onto the AC–MgFe composite. An increase in the adsorption temperature (25–45 °C) resulted in an improvement in the adsorption capacity of phenol (138.69–141.64 mg/g), indicating a favorable adsorption at elevated temperatures. Based on the Langmuir model assumption, the sorption of phenol molecules onto AC–MgFe was predominately via strong chemical interaction forces comprising the interaction of phenol molecules with AC–MgFe functional groups (OH, C=O, C-O, and MMO).

### 3.4. Phenol Adsorption Thermodynamic

The energetic variations of phenol adsorption onto AC–MgFe composite were further evaluated via measurement of changes in the Gibbs free energy (ΔG, kJ/mol), enthalpy (ΔH, kJ/mol), and entropy (ΔS, kJ/mol) at three tested temperatures (25–45 °C). The respective thermodynamic parameters were estimated using the following Equations (1) and (2) and are summarized in Table 5:

(1) $$\Delta G=-RTlnK_{d},$$

(2) $$lnk_{d}=\frac{\Delta S}{R}-\frac{\Delta H}{RH},$$

where constant *K_d_* is the thermodynamic equilibrium constant that was calculated using the method of Xin et al. [39] by plotting ln(q_e_/C_e_) vs. q_e_ and extrapolating q_e_ to zero; and R is the universal gas constant (8.314 J/mol/K).

Table 5 and Figure 7d demonstrate that an increase in temperature from 25 to 45 °C led to an increase in the values of ΔG from (−0.39 to −0.16) kJ/mol for AC–MgFe composite. The negative values of ΔG indicate that the phenol adsorption by AC–MgFe composite was favorable. The positive values of ΔH (3.85) kJ/mol and ΔS (0.011) kJ/mol-K for AC–MgFe composite demonstrated the phenol adsorption system is endothermic in nature and improved the magnitude of randomness at the solid–liquid interface, respectively [40].

### 3.5. Reusability of AC–MgFe Composite

**Figure 8:**
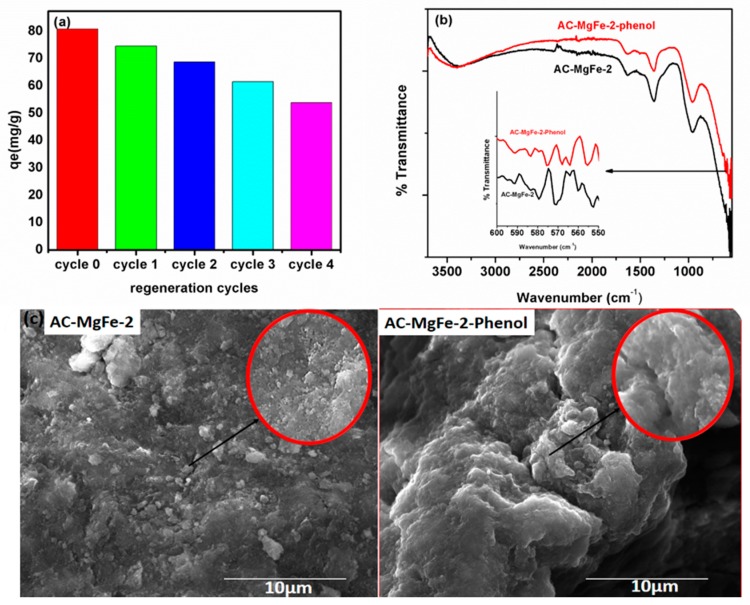
(**a**) Reusability performance of AC–MgFe after regeneration (**b**) FTIR and (**c**) SEM images of AC–MgFe-2 composite before and after phenol removal (red circle indicate magnified point image).

The recyclability of adsorbent is an important factor to demonstrate its potential for effective applications commercially. As such, the recyclability of the new AC–LDH was evaluated via adsorption-desorption experiments for up to four cycles. The spent AC–MgFe composite (100 mg) was regenerated via agitation of its slurry in a 100-mL 95% ethanol solution for 4 h. The results displayed in Figure 8a indicate that for up to two regeneration cycles, the adsorption capacity of AC–MgFe-2 declined by only 13.03% from 80.22 to 69.72 mg/g. After the fourth regeneration, the adsorption capacity further reduced down to 56.12 mg/g, which was still comparatively greater than that of some of the previously reported adsorbents (Table 4). This further confirmed that the synthesized AC–MgFe composite in this study exhibit excellent capability for the effective removal of phenol from wastewater streams.

### 3.6. Adsorption Mechanism of Phenol Onto AC–MgFe Composite

To further understand the adsorption interactions of phenol with the AC–MgFe composite surface, characterization (FTIR and SEM) of spent AC–MgFe-2 after phenol adsorption was performed. As depicted in the FTIR spectra (Figure 8b), the peaks correspond to C=N or C=O and C-O-C or C-O at 1609 and 953 cm^−1^ shifted to a lower intensity after phenol adsorption. Likewise, the broadband assigned to OH group vibrations at 3422 cm^−1^ showed changes in the wavenumber in the AC–MgFe–phenol spectra. Likewise, after phenol adsorption, the peaks below 600 cm^−1^ corresponding to MMO showed a noticeable alteration in the peak intensities and wavenumber. These FTIR results of the spent AC–MgFe composite support that the presence of functional groups on the surface of the AC–MgFe-2 composite have a strong interface with phenol molecules and act as active sorption sites to effectively remove phenol from the aqueous phase.

The SEM image of AC–MgFe-2 before and after phenol adsorption is shown in Figure 8c. The SEM image shows that the porous and coarse surface morphology with particles of various sizes of AC-MgFe-2 has completely transformed into a smooth surface with no pores, confirming that the entire composite surface was covered and saturated with phenol molecules. Therefore, based on the kinetics, isotherms, thermodynamics, and FTIR results (before and after phenol adsorption), it can be inferred that the adsorption of phenol molecules onto AC–MgFe-2 is led by multiple mechanisms attributed to the synergetic effect of activated carbon and MgFe LDH. Hence, chemical bonding with hydroxyl groups and mixed metal oxides and internal pore diffusion via π–π interactions could be the main dominating mechanism of the phenol–AC–MgFe adsorption system.

### 3.7. Comparison with Other Adsorbents

Comparing the sorption capacity of synthesized activated-carbon decorated MgFe composite via the co-precipitation method with the sorption capacity of other studied materials for phenol removal (Table 4) clearly confirmed that the AC–MgFe is a highly promising adsorbent. The adsorption capacity of AC–MgFe is higher compared to other LDHs and its derivatives, signifying that the coupling of activated carbon onto layers of MgFe is a favorable approach to enhanced removal of phenol from wastewater.

## 4. Materials and Methods

### 4.1. Materials

ZnCl_2_ sewage-based activated carbon was synthesized as per our previous study reported elsewhere [17] from the sewage sludge of a wastewater treatment plant located in Dhahran, Saudi Arabia. All the purchased materials were high purity (99.99%) and used without any modification. They included iron (III) nitrate nonahydrate [Fe(NO_3_)_3_·9H_2_O] and magnesium (II) nitrate hexahydrate [Mg(NO_3_)_2_⋅6H_2_O], and phenol purchased from Sigma Aldrich Co. (USA). The 500 mg/L stock solution of phenol was prepared and diluted to the required concentrations (20–120 mg/L) by using double distilled water.

### 4.2. Syntheses of ZnCl_2_-Activated Carbon Decorated MgFe LDH (AC–MgFe) Composites

The ZnCl_2_-activated carbon–MgFe LDH (AC–MgFe) composites were produced using the co-precipitation technique. Initially, a precise amount of ZnCl_2_-activated carbon corresponding to the MgFe amount ratio (Table 6) was ultrasonicated for 30 min in 50 mL of distilled water to obtain a homogenous dispersion. Simultaneously, 4.04 g of ferric (0.1 M) and 2.54 g (0.1 M) of magnesium nitrate salts were dissolved in 100 mL of distilled water in a reaction flask equipped with a magnetic stirrer. The solution was stirred vigorously for 15 min at 90 °C. Later, the pH of the solution was adjusted to 10 ± 0.5 using 1 M NaOH. After achieving the required pH, the reactor was then subjected to refluxing at 90 °C for 18 h. The resulting product was centrifuged and washed with DI water, followed by ethanol washing for the removal of unreacted salts and impurities. The final product was then dried at 85 °C in an oven for 48 h. The powder AC–MgFe composites were then stored in a desiccator for the adsorption experiments.

### 4.3. AC–MgFe Composites Characterization

The surface properties of the produced AC–MgFe composites were investigated via different techniques that included Fourier transform-IR (FTIR, Nicolet 6700, resolution 4 cm^−1^), X-ray diffraction (XRD, D8 advance X-ray instrument, wavelength = 0.1542 nm, and 2θ = 10° to 80°), scanning electron microscopy (SEM, SM-6460LV(Jeol)), transmission electron microscopy (FEI, Morgagni 268, Berno Czech republic), and Brunauer Emmett Teller (BET, Micromeritics, Tristar II series).

### 4.4. Phenol Uptake from Water Experiment

Initially, 20 mg/L of phenol solution at pH range 2–12, was agitated with 0.01 g of each AC–MgFe composite for 24 h at 275 rpm and 25 °C, respectively in 50-mL flat-bottomed plastic vials. Based on the pH results, the composite showing a higher removal efficiency of phenol at a certain pH was selected, and detailed adsorption experiments were further conducted for evaluation of the influence of adsorption parameters, such as the adsorbent amount, initial phenol concentration, and temperature and contact time, via equilibrium and kinetics studies. Accurately, 30 mL of phenol solution (20–120 mg/L) containing 0.01 g of the selected AC-MgFe composite in 50-mL plastic vials was agitated for 360 min at 275 rpm, and temperature range of 25 to 45 °C. The requisite pH of the phenol solution was adjusted using 0.1 mol/L HCl and 0.1 mol/L NaOH solutions. After agitation, the mixture was centrifuged and then filtered using 0.45-µm filter paper (cellulose acetate), and the residual phenol concentration was estimated using high-performance liquid chromatography (Thermo-scientific) equipped with a UV detector. The adsorption experiments were conducted in duplicates and the average values are indicated in the result section.

The amount of the phenol adsorbed on the AC–MgFe q_e_ (mg/g), and percentage removal efficiency were estimated according to Equations (3) and (4), respectively:

(3) $$\mathrm{Adsorption}\;\mathrm{capacity}=q_{e}=\frac{\left(c_{0}-c_{e}\right)v}{w},$$

(4) $$\mathrm{Percentage}\;\mathrm{removal}=\frac{\left(c_{0}-c_{e}\right)}{c_{e}}\times 100,$$

where *C_o_* and *C_e_* are the initial and equilibrium concentration (mg/L) of phenol in solution, respectively, *q_e_* (mg/g) is the equilibrium adsorption capacity, W (g) is the weight of the AC–MgFe composite, and V (L) is the volume of the solution.

### 4.5. Evaluation of Phenol Sorption Mechanism onto Activated Carbon–MgFe Composite

To clearly understand the sorption behavior of phenol molecules by the AC–MgFe composite, kinetic, isotherm, and thermodynamic models were applied to analyze the obtained phenol adsorption data. Four kinetic models, including pseudo-first and pseudo-second-order, and Elovich, and intraparticle diffusion, were fitted to the kinetic data to determine the actual rate-limiting step and dominant interactions (physical, chemical, pore diffusion, etc.) involved in the phenol AC–MgFe adsorption system. The linear forms of the kinetic models and their estimated parameters are listed in Table 3. The isotherm models, namely the Langmuir and Freundlich isotherms, were applied to the equilibrium data to understand the sorption phenomena of the adsorbate (phenol) onto the adsorbent (AC–MgFe composite) surface. Their respective linear equations and calculated parameters are summarized in Table 4.

## 5. Conclusions

The present study demonstrated a new effective adsorbent, produced via decoration of sewage sludge-based activated carbon onto layers of MgFe layered double hydroxides, for the improved removal of phenol from the water phase. The new composites were obtained at different mass ratios of AC and MgFe LDH via the simple co-precipitation method. The characterization of composites confirmed that the embedding of activated carbon with MgFe resulted in a significant improvement in the surface area and oxygen functionalities without damaging the crystalline structure. Accordingly, the composite exhibited an abundance of sorption active sites, leading to higher removal of phenol. The isotherm and kinetic data were appropriately fitted by the Langmuir and pseudo-second-order model, respectively. The maximum adsorption capacity of phenol exhibited by the AC–MgFe composite was 139.69 mg/g, which is comparatively higher than adsorbents reported in the literature, attributed to the synergic effect of activated carbon and MgFe, facilitating efficient uptake of phenol molecules from the aqueous phase. The phenol adsorption mechanism onto AC–MgFe is largely governed by multiple interaction forces involving chemical reactions with hydroxyl, carboxyl, and mixed metal oxide groups and pore penetration phenomena via π–π interactions. The AC–MgFe showed excellent removal after four regeneration cycles, which is evidence of its potential for an effective phenol removal process.

## Figures and Tables

**Table 1:** Surface area, pore volume, and pore size of AC–MgFe composites.

	AC–MgFe-1	AC–MgFe-2	AC–MgFe-3
BET surface area (m^2^/g)	257.22	233.76	168.92
Pore volume (cm^3^/g)	0.23	0.27	0.21
Micropore volume (DR method) (cm^3^/g)	0.18	0.18	0.17
Pore diameter (based on BJH) (nm)	3.36	3.36	3.42

**Table 2:** Parameters of pseudo-first, pseudo-second-order, elovich, and intra particle diffusion kinetic models for phenol removal onto AC–MgFe-2 composite.

Adsorbent	C_o_		Pseudo First Order $ln\left(q_{e}-q_{t}\right)=lnq_{e}-k_{1}t$			Pseudo Second Order $\frac{t}{q_{t}}=\frac{t}{q_{e}}+\frac{1}{k_{2}q_{e}^{2}}$			
		**q_e (exp)_**	**q_e_**	**k_1_**	**R^2^**	**q_e_**	**k_2_ × 10^−2^**	**h**	**R^2^**
AC-MgFe-2	20	14.85	35.46	0.09	0.826	19.44	0.27	0.60	0.997
	60	40.68	280.81	0.10	0.731	54.05	0.24	0.71	0.992
	100	54.3	247.2	0.09	0.836	74.62	0.17	0.94	0.987
			**Elovich** $q_{t}=\frac{1}{\beta }ln\left(\alpha \beta \right)+\frac{1}{\beta }ln\left(t\right)$			**Intra particle diffusion** **q_t_ = *k*_d_ t^1/2^ + C**			
		**q_e (exp)_**	**α**	**β**	**R^2^**	**K_p_**	**C**	**R^2^**	
AC-MgFe-2	20	14.85	2.25	0.06	0.967	0.71	3.79	0.799	
	60	40.68	1.71	0.08	0.977	2.82	0.04	0.966	
	100	54.3	1.39	0.33	0.952	3.95	0.71	0.933	

**Table 3:** Parameters of linear Langmuir and Freundlich isotherm models for phenol adsorption onto AC–MgFe-2 composites.

Adsorbent	T (K)	Langmuir $\frac{C_{e}}{q_{e}}=\frac{q_{m}}{K_{l}}+\frac{C_{e}}{q_{m}}$				Freundlich $lnq_{e}=lnK_{F}+\frac{1}{n}lnC_{e}$			
		**q_max_** **(mg/g)**	**K_L_**	**R^2^**	**RMSE**	**K_F_**	**1/n**	**R^2^**	**RMSE**
AC-MgFe-2	298	138.69	0.003	0.997	0.001	4.01	1.26	0.996	0.046
	308	139.77	0.004	0.994	0.001	3.70	1.25	0.996	0.047
	318	141.64	0.004	0.990	0.002	3.48	1.25	0.996	0.049

**Table 4:** Adsorption capacity and parameters of phenol onto recently studied adsorbents.

Adsorbent	pH	Time (Minutes)	Adsorption Capacity (mg/g)	References
Iron impregnated activated carbon	7	90	20	[35]
Diethylenetriamine-modified activated carbon	3	120	18.12	[36]
milled MgAl	-	180	82.6	[37]
Aliquat 336 functionalized Zn-Al	6.5	60	64.7	[38]
Calcined MgAl/SWCNT	6	3600	219.0	[23]
AC-MgFe composite	6	180	138.69	This work

**Table 5:** Thermodynamic parameters of phenol adsorption onto AC–MgFe composites.

	T (K)	∆G (kJ/mol)	∆H (kJ/mol)	∆S (kJ/mol K)
AC–MgFe-2	298	−0.39		
	308	−0.32	3.85	0.011
	318	−0.16		

**Table 6:** Composition of AC–MgFe composites.

Sample	AC (g)	Mg:Fe Salts (g) (0.1:0.1)M
AC–MgFe-1	0.5	2.54:4.04
AC–MgFe-2	0.25	2.54:4.04
AC–MgFe-3	0.1	2.54:4.04

## Supplementary Materials

Supplementary materials can be found at https://www.mdpi.com/1422-0067/21/5/1563/s1.
