# Supplementary figures and images for: Sewage Sludge ZnCl2-Activated Carbon Intercalated MgFe–LDH Nanocomposites: Insight of the Sorption Mechanism of Improved Removal of Phenol from Water
Source: Int J Mol Sci. 2020 Feb 25;21(5):1563. doi: 10.3390/ijms21051563 (PMC7084656; doi:10.3390/ijms21051563)

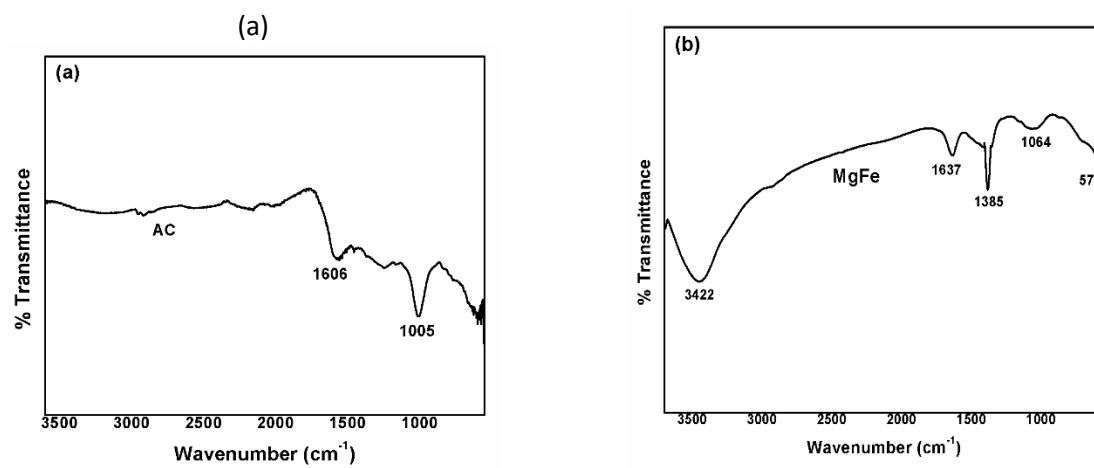

Figure S1. FTIR of AC (a) and MgFe (b)

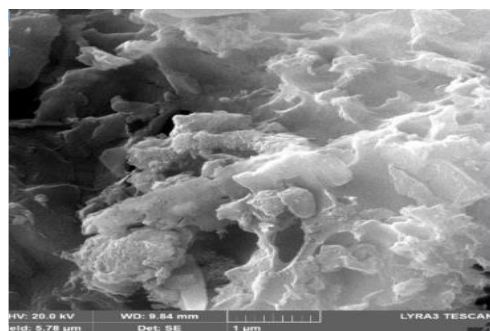

Figure S2: SEM micrographs of the Zn-SBAC

Supplement: Supplementary file 1 [file ijms-21-01563-s001.pdf]
